# Diagnosing anisakiasis in the emergency department

**DOI:** 10.1186/cc12209

**Published:** 2013-03-19

**Authors:** T Takabayashi, S Ishimatsu, N Otani, T Mochizuki, R Miyamichi

**Affiliations:** 1 St. Luke's International Hospital, Tokyo, Japan

## Introduction

Humans can be incidentally parasitized by third-stage Anisakis larvae after ingestion of raw or undercooked seafood. Although the clinical symptom of anisakiasis is abdominal pain, the clinical finding is nonspecific and may be misdiagnosed as appendicitis, ileus, and so forth. Every year, >40,000 patients visit our ED and our hospital near the Tsukiji market (famous seafood market in Tokyo). We often observed cases of anisakiasis in patients who visited the hospital with abdominal pain as the chief complaint. Thus, we researched to determine factors useful to diagnose anisakiasis.

## Methods

We retrospectively reviewed data of 83 patients (58 men, 25 women) diagnosed with anisakiasis in our ED (22 July 2003 to 22 July 2012) and examined the usefulness of clinical history, blood test, diagnostic imaging, and so forth, for anisakiasis diagnosis. Diagnosis was made after (A) endoscopically proven Anisakis polypide and (B) a hematologically positive Anisakis antibody (IgG, IgA) and CT diagnostic imaging.

## Results

Of the 83 patients, 39 had gastric anisakiasis and 44 had small intestinal anisakiasis. All gastric and small intestinal anisakiasis patients were diagnosed by methods A and B, respectively. A blood test was unable to show the specific inflammatory reaction. A history of raw or undercooked seafood ingestion was noted in 95.2% (79/83) of the patients. This was observed in 100% (39/39) of the gastric anisakiasis patients and 91% (40/44) of the small intestinal anisakiasis patients. With regard to the development of symptoms, symptoms for gastric anisakiasis developed within 48 hours and reached a peak in less than 6 hours, whereas the symptoms for small intestinal anisakiasis reached a peak in 48 hours and persisted for a maximum of 5 days. Diagnostic CT imaging revealed that all the patients with gastric anisakiasis demonstrated edematous wall thickening of gastric mucosa, and ascites was observed in 44.4% (12/27) of these patients. The patients with small intestinal anisakiasis demonstrated limited edematous wall thickening of the intestinal tract, and ascites was observed in 90.9% (40/44) of these patients. Furthermore, phlegmon of mesentery fat was observed in 72.7% (32/44) of the small intestinal anisakiasis patients.

## Conclusion

When the cause of abdominal pain cannot be determined by initial assessment, anisakiasis should be considered, especially if the patient has a history of raw or undercooked seafood ingestion. In the ED, certain methods of diagnosis are evaluation of the time to develop symptoms and CT imaging, and a history of raw or undercooked seafood ingestion should be considered in the diagnosis.
